# A genetically inducible porcine model of intestinal cancer

**DOI:** 10.1002/1878-0261.12136

**Published:** 2017-10-10

**Authors:** Morten M. Callesen, Sigrid S. Árnadóttir, Iben Lyskjær, Mai‐Britt W. Ørntoft, Søren Høyer, Frederik Dagnæs‐Hansen, Ying Liu, Rong Li, Henrik Callesen, Mads H. Rasmussen, Martin F. Berthelsen, Martin K. Thomsen, Pawel J. Schweiger, Kim B. Jensen, Søren Laurberg, Torben F. Ørntoft, Jannik E. Elverløv‐Jakobsen, Claus L. Andersen

**Affiliations:** ^1^ Department of Molecular Medicine Aarhus University Hospital Denmark; ^2^ Department of Pathology Aarhus University Hospital Denmark; ^3^ Department of Biomedicine Aarhus University Denmark; ^4^ Department of Animal Science Aarhus University Denmark; ^5^ Biotech Research and Innovation Centre University of Copenhagen Denmark; ^6^ Surgical Department P Aarhus University Hospital Denmark

**Keywords:** inducible intestinal cancer, oncopig, tissue‐specific activation, transgenic porcine model

## Abstract

Transgenic porcine cancer models bring novel possibilities for research. Their physical similarities with humans enable the use of surgical procedures and treatment approaches used for patients, which facilitates clinical translation. Here, we aimed to develop an inducible oncopig model of intestinal cancer. Transgenic (TG) minipigs were generated using somatic cell nuclear transfer by handmade cloning. The pigs encode two TG cassettes: (a) an Flp recombinase‐inducible oncogene cassette containing KRAS‐G12D, cMYC, SV40LT – which inhibits p53 – and pRB and (b) a 4‐hydroxytamoxifen (4‐OHT)‐inducible Flp recombinase activator cassette controlled by the intestinal epithelium‐specific villin promoter. Thirteen viable transgenic minipigs were born. The ability of 4‐OHT to activate the oncogene cassette was confirmed *in vitro* in TG colonic organoids and *ex vivo* in tissue biopsies obtained by colonoscopy. In order to provide proof of principle that the oncogene cassette could also successfully be activated *in vivo*, three pigs were perorally treated with 400 mg tamoxifen for 2 × 5 days. After two months, one pig developed a duodenal neuroendocrine carcinoma with a lymph node metastasis. Molecular analysis of the carcinoma and metastasis confirmed activation of the oncogene cassette. No tumor formation was observed in untreated TG pigs or in the remaining two treated pigs. The latter indicates that tamoxifen delivery can probably be improved. In summary, we have generated a novel inducible oncopig model of intestinal cancer, which has the ability to form metastatic disease already two months after induction. The model may be helpful in bridging the gap between basic research and clinical usage. It opens new venues for longitudinal studies of tumor development and evolution, for preclinical assessment of new anticancer regimens, for pharmacology and toxicology assessments, as well as for studies into biological mechanisms of tumor formation and metastasis.

**Abbreviations**
4‐OHT: 4‐hydroxytamoxifen
BFP: blue fluorescent protein
CAG: cytomegalovirus early enhancer chicken β‐actin rabbit β‐globin
CMV: cytomegalovirus
ddPCR: digital droplet polymerase chain reaction
DOX: doxycycline
FAP: familial adenomatous polyposis
Flp‐ERT2: flippase estrogen receptor type 2FRTFlp recombinase recognition target sites
HRP: horseradish peroxidase
IHC: immunohistochemistry
LDI‐PCR: long‐distance inverted PCR
NET: neuroendocrine tumor
P/S: penicillin/streptomycin
RFP: red fluorescent protein
ROX: Dre recombinase recognition target sites
rtTR‐KRAB: reverse tetracycline repressor‐fused Krüppel‐associated box
SCNT: somatic cell nuclear transfer
SV40LT: simian virus‐40‐large T
TG: transgenic
TRE: Tet‐responsive elements
WT: wild‐type
YFP: yellow fluorescent protein

## 1 Introduction

In cancer research, animal models play an important role for establishing a link between basic research and clinical application. Numerous rodent models have been developed for studying intestinal cancer. They have provided insights into pathogenic mechanisms, served as tools for discovery and validation of novel therapeutic targets and for testing chemoprevention strategies (Le Magnen *et al*., 2016). However, no rodent model fully mimics human intestinal carcinogenesis (Washington *et al*., 2013; Young *et al*., 2013). The limitations of rodents as models of human cancer include large differences in lifespan, size, and physiology. To augment studies in mice, new animal models of cancer are needed. The pig is a promising alternative model organism due to its high degree of similarity to humans, including longevity, size, organ anatomy, physiology, drug metabolism, genetics, and immunology (Flisikowska *et al*., 2013; Forster *et al*., 2010; Kuzmuk and Schook, 2011; Manno *et al*., 2016; Mote and Rothschild, 2006; Wernersson *et al*., 2005). Importantly, the large size of pigs also brings opportunities for longitudinal sampling and therefore monitoring of tumor evolution during treatment, which is generally not possible in rodents.

Throughout the industrialized countries, intestinal cancers are among the most common malignancies (Siegel *et al*., 2016). The molecular mechanisms underlying intestinal oncogenesis is well described (Fearon and Vogelstein, 1990; Wood *et al*., 2007), which is essential for generating relevant transgenic (TG) animal models. Cancers arising from the intestinal epithelium are believed to start as benign adenomatous polyps, a process often initiated by the activation of canonical Wnt signaling, that enables the cells to avoid the normal differentiation and exfoliation from the intestinal wall (Krausova and Korinek, 2014). Most commonly, Wnt is activated through mutational inactivation of the tumor suppressor gene APC leading to upregulation of downstream Wnt targets, such as cMYC (Finch *et al*., 2009). A pig model of human familial adenomatous polyposis (FAP) syndrome was recently reported (Flisikowska *et al*., 2012). In line with humans, the FAP model developed multiple benign polyps; however, so far no malignant tumors have been reported (Flisikowska *et al*., 2012). Other recent oncopig models addressed human Li‐Fraumeni syndrome (Sieren *et al*., 2014). In one model, the oncopigs were homozygous for the TP53‐R167H mutation and developed numerous tumors throughout the body in resemblance with the human disease (Leuchs *et al*., 2012). Another model allowed for more controllable tumor formation – spatial and temporal – by encoding Cre recombinase‐inducible TP53‐R167H and KRAS‐G12D (Schook *et al*., 2015). Upon injection of Cre‐expressing adenovirus, these pigs developed various mesenchymal tumors at the injection site. Common for the two models are their lack of tissue specificity and the formation of tumors of multiple cellular origins.

Here, we present a novel oncopig model with inducible intestinal‐specific carcinogenesis leading to rapid formation of metastatic carcinoma. TG minipigs were created by porcine somatic cell nuclear transfer (Du *et al*., 2005; Schmidt *et al*., 2010). The model encodes two TG cassettes: (a) a flippase (Flp) recombinase‐inducible oncogene cassette containing KRAS‐G12D, cMYC, and simian virus‐40‐large T (SV40LT) antigen and (b) a 4‐hydroxytamoxifen (4‐OHT)‐inducible Flp recombinase activator cassette (Flp‐ERT2) controlled by the villin promoter, which is specifically expressed in the epithelium of the intestinal mucosa (El Marjou *et al*., 2004; Kucherlapati *et al*., 2006; Madison *et al*., 2002). The model enables induction of intestinal carcinogenesis through the activation of KRAS and cMYC oncogenic signaling and through SV40LT‐mediated inactivation of the tumor suppressors, p53 (Bargonetti *et al*., 1992; Lilyestrom *et al*., 2006) and pRB (Resnick‐Silverman *et al*., 1991). This oncopig model represents an exciting disease model for intestinal cancer, as it opens a range of possibilities for longitudinal studies of intestinal cancer development, metastatic disease, and therapy response.

## 2 Materials and methods

All animal experiments were conducted and approved by the Danish Animal Experiments Inspectorate (license no. 2012‐15‐2934‐00031 and 2014‐15‐0201‐00433) and adhered to ARRIVE. The Göttingen Ellegaard minipigs (Ellegaard Göttingen Minipigs A/S, Denmark) were housed and cared for in accordance with the Danish Animal Research Proposal on genetically modified animals and the EU Directive 2010/63/EU for animal experiments.

### 2.1 Cassette construction

The oncogene cassette was constructed in multiple cloning steps. The following plasmids were used in the construction: the pLVCT‐rtTR‐KRAB‐2SM2 (Addgene plasmid #11779), and the pBABE‐puro‐SV40LT (Addgene plasmid #13970); the YFP‐F2A‐KRAS (isoform‐B) G12D‐T2A‐cMYC, CAG‐RFP, and the TRE element were ordered from GenScript (Piscataway, NJ, USA). The activator cassette was constructed from the following plasmids: 12.4‐kb murine villin promoter (Addgene plasmid #19358), blue fluorescent protein (BFP) mTurquoise2 (Addgene plasmid #54842) (Goedhart *et al*., 2012), PGK‐FlpO (codon optimized), and Cre‐ERT2 (gifts from Frank Schnütgen, Goethe University, Germany). Both cassettes included left and right inverted repeats (LIR and RIR) and LoxP elements from the floxed‐Ei‐Ubi‐*PSEN1M146I* plasmid described in Jakobsen *et al*. (2013). The oncogene and activator cassette are available from Addgene (deposited as Addgene plasmid #67277 and #67278, respectively).

### 2.2 Transfection and selection of cell clones

Fibroblasts from female neonate minipig ear notch biopsies were cultured in DMEM (Lonza, Basel, Switzerland), supplied with 15% FBS, 1% penicillin/streptomycin (P/S), and 1% glutamine (Sigma‐Aldrich, St. Louis, MO, USA). Fibroblasts at 50–60% confluence were cotransfected with 40 ng circular Sleeping Beauty 100X (Mátés *et al*., 2009), 560 ng oncogene, and 3400 ng activator cassette (1 : 7) using 6 μL TurboFect lipofectamine (Thermo Fisher Scientific, Waltham, MA, USA). Puromycin (1.6 μg·mL^−1^ – 14 days)‐resistant colonies were isolated and expanded with bovine FGF supplied (14 ng·L^−1^). RFP^+^/BFP^−^ colonies validated by PCR to contain both the TG cassettes were used for cloning.

### 2.3 Handmade cloning (HMC), embryo culture, and transfer

HMC was performed as described by Kragh *et al*. (2004, 2009) with improvement by Du *et al*. (2005) using oriented, bisected, enucleated oocytes with partially digested zonae pellucidae. In brief, serum‐starved cell clones were attached 1 : 1 with a cytoplast and fused (BTX microslide 0.5 mm fusion chamber, model 450 BTX San Diego, US). Each cytoplast–TG fibroblast pair was fused with a second cytoplast creating a reconstructed embryo. Reconstructed embryos were incubated and cultivated for 5–6 days before surgically transferred into Danish landrace surrogate sows (Du *et al*., 2007; Schmidt *et al*., 2010). The farrowing was hormonally initiated at day 114 by intramuscularly injected prostaglandin, resulting in 13 viable TG minipigs.

### 2.4 Southern blot analysis

Genomic DNA (gDNA) was isolated from chorionic villi‐lysed neonatal ear biopsies, and Southern blotting of 6 μg gDNA, digested by 1.5 U·μg^−1^ DNA *Eco*RV and *Bst*Z17I (Thermo Fisher Scientific), on nitrocellulose membrane was performed as described (Jakobsen *et al*., 2011). The RFP and Flp DNA probes (705 and 777 bp) were purified from restriction‐cleaved plasmid DNA and labeled with ^32^P‐dCTP EasyTide (Perkin Elmer, Waltham, MA, USA) using the Prime‐It Random Labeling Kit (Agilent Technologies, Santa Clara, CA, USA). X‐ray films were developed at −70 °C for 48–72 h.

### 2.5 Long‐distance inverted PCR (LDI‐PCR)

LDI‐PCR was performed as previously described (Al‐Mashhadi *et al*., 2013). In brief, gDNA isolated from fetal TG fibroblasts was digested with *Bsu*36I and *Pac*I (Thermo Fisher). DNA was circularized by T4 ligase (Thermo Fisher) and used as template in a two‐round nested PCR setup to produce flanking genomic sequence. The DNA was Sanger‐sequenced and the upstream genomic sequence was mapped to the pig genome (Groenen *et al*., 2012) (S. scrofa 10.2). Primers are listed in Table S1.

### 2.6 IVIS optical imaging of organ fluorescence

*Ex vivo* epifluorescence imaging of pig organs was performed with the IVIS^®^ spectrum system (Perkin Elmer). Laser and filter settings for RFP (570/640 nm, 20 nm), YFP (500/540 nm, 20 nm), and BFP (430/500 nm, 20 nm) were applied. Fixed illumination settings (voltage, f/stop, field of view, and binning) were used, and fluorescence emission was normalized to photons per second per square centimeter per steradian over lamp watt per square centimeter [p·s^−1^·cm^−2^·sr^−1^]/[μW·cm^−2^] designated as mean radiant efficiency. The adaptive fluorescent background and tissue autofluorescence were subtracted in spectral unmixing and only photon counts > 600 were analyzed. Image and data analyses were performed with living image 4.3 (Perkin Elmer). The WT organs were coimaged to set autoexposure according to the brightness of the tissue and to automatically reduce false‐positive signal.

### 2.7 Quantitative PCR

RT‐qPCR and qPCR were performed using SYBR Green I Master Mix (Roche, Basel Switzerland) according to the manufacturer's instructions. All RT‐qPCR and qPCR measurements were made on a LightCycler 480 (Roche). The BFP and RFP allelic copy numbers were estimated from TG ear notch biopsy DNA and normalized to porcine GLIS3. Total RNA from fibroblast and fresh frozen tissue was purified using Maxwell^®^ 16 LEV simplyRNA (Promega, Madison, WI, USA) according to the manufacturer's guidelines. cDNA was synthesized using iScript™ Select cDNA Synthesis Kit (Bio‐Rad, Hercules, CA, USA). No‐RT controls were included to identify and exclude samples with contaminating gDNA. Relative expression levels were determined using the comparative *C*_t_ method (Livak and Schmittgen, 2001) and normalized to a geometric mean of the three reference genes *RPL4*,* HPRT1*, and *TBP* (Nygard *et al*., 2007; Vandesompele *et al*., 2002). Primer sequences are shown in Table S1.

### 2.8 Flp recombination validation by PCR and digital droplet PCR (ddPCR)

For Flp recombination validation by PCR, 20 ng of gDNA was PCR‐amplified with Q5^®^ Hot Start High‐Fidelity polymerase (NEB, MA, USA), 200 μm dNTP, 10 pmol forward (a) and 10 pmol reverse primer (b) multiplied in 35 cycles PCR (*T*_an._ 62 °C, 15 s and *T*_ex._ 72 °C 1 min 10 s).

Droplet digital PCR (ddPCR) quantification of allelic copy numbers was performed on a QX100™ ddPCR system according to the manufacturer's instructions (Bio‐Rad). TaqMan assays for quantifying the porcine genome (3–4), the oncogene cassette (5–6), and the Flp‐activated oncogene cassette (7–8) were profiled. The ddPCR data were analyzed using QuantaSoft™ (Bio‐Rad). Primer and probe sequences are listed in Table S1.

### 2.9 Flow cytometry

Puromycin‐resistant colonies were harvested and analyzed on an LSRFortessa flow cytometer (BD Bioscience, NJ, USA). Single‐cell gating and background fluorescent subtraction were applied before evaluation of the fluorescence signal. Laser and filter settings for RFP (561/630 nm, 22 nm), YFP (488/530 nm, 30 nm), and BFP (405/442 nm, 46 nm) were applied. To account for fluorescence spillover, negative controls, fluorescent minus one, and single reporter positive controls were analyzed for all reporter combinations. Results were analyzed using flowjo 10.0.7 (Tree Star Inc., Ashland, OR, USA).

### 2.10 Western blotting

Protein extraction and western blotting was performed using standard procedures. In brief, 30 μg protein per sample was separated by Bolt^®^ Bis‐Tris 4–12% SDS/PAGE (Thermo Fisher) and blotted onto polyvinylidene fluoride (PVDF) by iBlot^®^ (Invitrogen, Carlsbad, CA, USA) and blocked by 4% skim milk in 2% Tris‐buffered saline/Tween. Primary antibodies used in this study were as follows: 2A peptide 1 : 2000 ABS31 (Millipore, Billerica, MA, USA), SV40LT 1 : 400 Pab416 (Abcam, Cambridge, UK), and β‐actin 1 : 10 000 AC‐15 (Abcam). Secondary horseradish peroxidase (HRP) antibodies used in this study were as follows: goat anti‐rabbit (AP156P; Millipore) and rabbit anti‐mouse (ab97046; Abcam).

### 2.11 Endoscopy

Pigs were given a single rectal enema of 45 mL Phosphoral (ATC‐code: A06AD) 1 h prior to endoscopy, which was performed with an Olympus GIF‐Q180 gastroscope. The pigs were anesthetized by (1 mL per 15 kg body mass) intramuscular injection of Zoletil 50 Vet. mixture (125 mg zolazepam, tiletamine, 125 mg ketamine, 125 mg xylazine, and 25 mg butorphanol). Postoperatively, the pigs were given analgesics and water and fed *ad libitum*.

### 2.12 4‐OHT treatment of porcine intestinal organoids and mucosa biopsies

Pig intestinal mucosa samples were collected and stored in 4 °C transport medium consisting of advanced DMEM/F12 with 1% P/S (Life Technologies, Carlsbad, CA, USA) and 2.5 μg·mL^−1^ amphotericin (Sigma‐Aldrich). The isolation of crypts for organoid formation was performed essentially as previously described (Sato *et al*., 2011), although with a modified chelation buffer of PBS supplemented with 30 mm EDTA (Life Technologies) and 0.5 mm DTT (Sigma‐Aldrich). Crypts were suspended in (1 : 2) PBS and growth factor‐reduced Matrigel (BD Biosciences, San Jose, CA, USA), which was polymerized for 30 min at 37 °C and covered in organoid medium containing advanced DMEM/F12, supplemented with 1% P/S, 10 mm HEPES, 50 ng·mL^−1^ mEGF, 1x GlutaMax (all from Life Technologies), 1 mm *N*‐acetylcysteine, 10 μm SB202190 (Sigma‐Aldrich), 100 ng·mL^−1^ noggin (PeproTech, Rocky Hill, NJ, USA), 500 nm A‐83‐01 (Tocris), 25% Wnt‐3A‐conditioned medium, and 25% R‐spondin1‐conditioned medium (Sato *et al*., 2011). Mouse L‐cells expressing Wnt‐3A were kindly provided by Hans Clevers, Hubrecht Institute, The Netherlands, whereas Hek293T cells expressing R‐spondin1 were kindly provided by Calvin Kuo, Stanford University, USA. The organoids were grown at 37 °C in a 5% CO_2_ humidified incubator.

For testing oncogene cassette activation, *in vitro* organoids and *ex vivo* intestinal biopsies were cultured in organoid medium and DMEM 1% P/S + 10% FBS, respectively. Activation was performed with 1 μm 4‐OHT (Sigma‐Aldrich) for a minimum of 24 h before cell lysis and DNA purification. An overview of the usage of minipigs is shown in Fig. S3.

### 2.13 Immunohistochemistry

Tissues sections (4 μm) fixed in 10% formaldehyde and embedded in paraffin received antigen retrieval at 100 °C in citrate buffer. Sections were blocked in 2.5% BSA in PBS + 0.1% Tween 20 and the following primary antibodies were used: synaptophysin (MRQ‐40), CD56 (MRQ‐42), CDX‐2 (EPR2764Y), Ki67 (30‐9) (Ventana Roche, Tucson, AZ, USA) and SV40LT (Pab416; Abcam). Secondary antibodies were coupled to HRP, and counterstaining was performed with hematoxylin. The proportion of positively stained cells was estimated using Fiji (Schindelin *et al*., 2012).

## 3 Results

### 3.1 Generation of TG intestinal cancer minipigs

**Figure 1:**
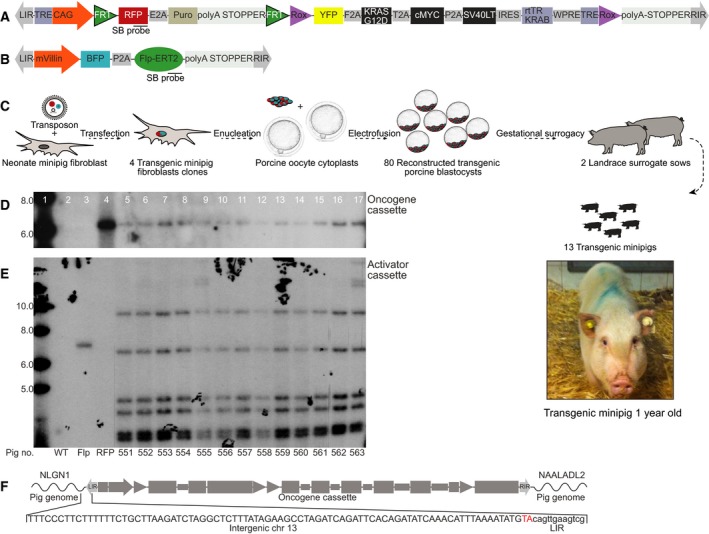
Genetic design of the porcine intestinal cancer model. (A) The inducible polycistronic oncogene cassette is a *Sleeping Beauty* transposon controlled by a CAG promoter. The cassette consists of two parts: The first part encodes a red fluorescent protein (RFP) reporter and a puromycin *N*‐acetyl transferase. Its transcription is terminated by a strong polyadenylation site, which prevents read‐through into the second part. Flp recombinase recognition target sites (FRT) flank the first part and enable excision. The second part consists of yellow fluorescent protein (YFP) and three oncogenes KRAS‐G12D, cMYC and SV40LT flanked by Dre recombinase recognition target sites (ROX). Expression from the oncogene cassette can be terminated by Dre recombination or inhibited using the doxycycline‐responsive element rtTR‐KRAB docking at the Tet‐responsive elements (TRE); (B) the FlpO‐inducible activator cassette is also a *Sleeping Beauty* transposon. Expression from the cassette is controlled by an intestinal‐specific villin promoter. The cassette encodes a blue fluorescent protein (BFP) reporter and a 4‐OHT‐inducible estrogen receptor‐type2‐conjugated Flp recombinase (Flp‐ERT2). Probe positions for Southern blotting are indicated in both cassette maps; (C) overview of the cloning procedure. *Sleeping Beauty* mediates stable integration of the cassettes, followed by porcine SCNT by handmade cloning and transfer of transgenic porcine blastocyst into surrogate landrace sows. Image of TG pigs at day 357; (D) Southern blot for quantification of cassettes in neonate TG minipig ear fibroblasts probed by either ^32^P‐dCTP RFP DNA probe or (E) ^32^P‐dCTP Flp DNA probe. Lanes 1–4 depict a 1‐kb ladder, WT fibroblast DNA as negative control, and WT fibroblast DNA spiked by either Flp activator‐ or oncogene cassette DNA as positive controls. Lanes 5–17 present genomic fibroblast DNA from 13 TG minipigs. High molecular bands in pigs no. 555 and 563 are likely artifacts. (F) Mapping of the genomic integration site of the oncogene cassette to a region between the genes *NLGN1* and *NAALADL2* on chromosome 13 using LDI‐PCR in TG minipig no. 556 fibroblasts.

TG primary neonate Göttingen minipig fibroblasts were generated by Sleeping Beauty transposase‐mediated integration of two cassettes into the pig genome: an oncogene and an activator cassette (Fig. 1A, B). The oncogene cassette consists of two parts driven by a ubiquitous CAG promoter. The first part consists of RFP and a puromycin selection gene followed by a polyA stop element, which prevents expression of the second part of the cassette. The first part is flanked by FRT sites and can be excised upon expression of Flp recombinase. Excision of the first part promotes expression from the second oncogene element. The polycistronic oncogene element consists of YFP and three oncogenes separated by 2A linker peptides: *KRAS‐G12D, WT cMYC,* and *SV40LT* (Fig. 1A). The activator cassette is driven by the intestinal‐specific villin promoter and encodes a BFP as well as a Flp recombinase fused to the triple mutant form of the human estrogen receptor (Flp‐ERT2), which does not bind its natural ligand (17β‐estradiol) at physiological concentrations, but will bind the estrogen receptor ligand 4‐OHT (Fig. 1B). In the absence of 4‐OHT, the Flp‐ERT2 fusion protein will be located in the cytoplasm and accordingly the Flp‐ERT2 is unable to mediate DNA recombination. However, in the presence of 4‐OHT, the fusion protein translocates to the nucleus and the Flp‐ERT2 recombinase activity becomes active (Brocard *et al*., 1997; Reinert *et al*., 2012). The intestinal cancer cell line CaCo2, primary fibroblast, and embryonic kidney cell line HEK293T were used to confirm tissue‐specific expression of the villin promoter (Fig. S1A) and to test its activity (Fig. S1B) compared with CAG and CMV promoters. Nuclear translocation and activation of the Flp‐ERT2 recombinase can be induced by treatment with 4‐OHT. The oncogene cassette furthermore includes two terminal tet‐responsive elements (TRE) and a reverse tetracycline repressor‐fused Krüppel‐associated box KRAB‐off element, enabling conditional inhibition of oncogene expression by doxycycline (DOX) (Groner *et al*., 2012; Szulc *et al*., 2006). Additionally, the oncogene cassette contains two Dre recombinase recognition sites (Rox), enabling termination of oncogene expression through excision (Fig. 1A). For cloning of TG minipigs, 18 puromycin‐resistant colonies were harvested. Of these, nine contained both cassettes. Four showed a single integrated oncogene cassette and multiple integrated activator cassettes (Fig. S2A). The latter are beneficial as a positive correlation is expected between the Flp level and the likelihood of activating the oncogene cassette. The five remaining clones were mixed populations of wild‐type (WT) and TG cells and were excluded from further analysis. As expected, the four selected clones expressed RFP, but not BFP or YFP (Fig. S2B). All four clones were expanded and forced into G_0_ by serum starvation, electrofused with enucleated oocytes, and cultured for 5–6 days. Two pools of cloned TG embryos containing either 80 blastocysts or 66 blastocysts + 14 morulas were prepared and transferred into two recipient landrace surrogate sows. The sows farrowed 13 TG minipigs, giving a total cloning efficiency of 8.9% from the initial blastocysts. Figure 1C illustrates the cloning procedure. An overview of how the 13 TG minipigs were used in this study is provided in Fig. S3. Southern blotting of the 13 TG minipigs revealed integration of one oncogene cassette and indicated six activator cassettes (Fig. 1D,E). Unexpectedly, all TG minipigs showed the same bands for both cassettes, indicating that they all originated from the same clone. Using LDI‐PCR, the integration site of the oncogene cassette was mapped to an intergenic region on chromosome 13 (13; 121620509–121520689) S. scrofa 10.2, between the genes *NLGN1* and *NAALADL2* (Fig. 1F). Subsequent genomic PCR spanning from the cassette and into the neighboring region on chromosome 13 identified the same integration site in all 13 minipigs (Fig. S2C), supporting that the 13 TG pigs originated from the same clone. Importantly, the resulting TG minipigs showed unaffected embryogenesis and development, indicating that the integration sites of the oncogene and activator cassettes in this clone did not implicate essential genes.

### 3.2 Tissue specificity of the activator and oncogene cassettes

**Figure 2:**
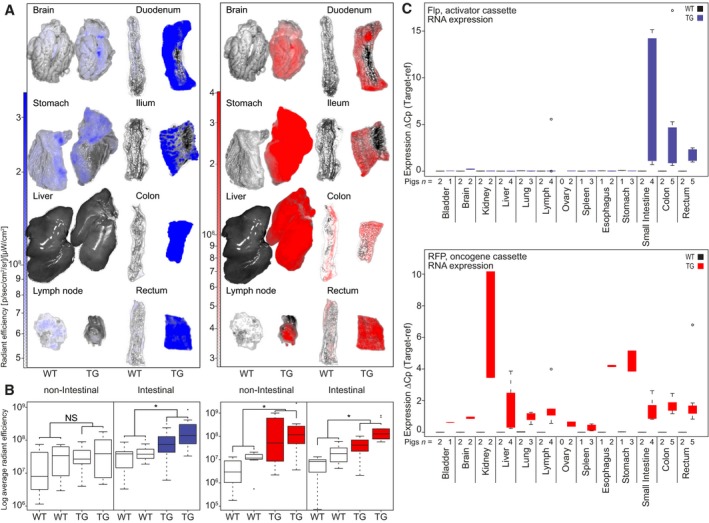
Fluorescence reporters and RT‐qPCR show intestinal‐specific expression of villin‐BFP and universal expression of CAG‐RFP. (A) IVIS analysis of isolated organs from TG minipigs (*n* = 2) and landrace WT pigs (*n* = 2) depicted are image overlays of bright field and fluorescence; (B) quantification of the IVIS‐collected data on nonintestinal and intestinal organs from two TG pigs and two WT landrace pigs (for images and raw values, Fig. S4). N.S. = not significant, *P <* *0.05. Boxplots indicate the interquartile range and median. Minimum/maximum values are represented by whiskers and outliers by dots; (C) RT‐qPCR‐based assessment of RFP and Flp expression in organs from TG pigs (*n* = 5). The expression was normalized to the geometric mean of the reference genes RPL4, HPRT, and TBP.

To assess the tissue specificity of the cassettes, the BFP, RFP, and YFP fluorescence in selected organs from untreated TG pigs (*n* = 2) was measured by IVIS analysis. WT animals (*n* = 2) were used as negative controls (Figs 2 and S4). The expression of BFP from the activator cassettes was primarily seen in the mucosa of the intestine, while RFP from the oncogene cassette was ubiquitously expressed in both intestinal (rectum, colon, ileum, jejunum, duodenum, and stomach) and nonintestinal organs (eye, brain, esophagus, heart, kidney, pancreas, bladder, liver, spleen, and lymph nodes) (Figs 2A,B and S4). We observed tissue autofluorescence from reflective and bright appearing organs (including eyes and brain) in both WT and TG animals that overlapped with the BFP emission spectrum, which is a well‐known phenomenon (Becker *et al*., 2001; Weissleder, 2001). Epifluorescence quantification revealed significantly higher intestinal BFP expression compared to nonintestinal *P* = 0.0004, and background (WT) (*P* = 0.0002, Wilcoxon signed‐rank test) (Fig. 2B). RFP expression was significantly higher in all organs of TG pigs compared to background (WT) (intestinal *P* = 1.07e‐7, nonintestinal *P* = 8.86e‐5). No difference in RFP expression was observed between intestinal and nonintestinal TG organs (*P* = 0.87). No YFP expression was observed in any TG organ, indicating that oncogene expression was efficiently prevented by the polyA stopper without any leakage (Fig. S4B). The expression pattern of the two cassettes was confirmed at the mRNA level by RT‐qPCR using organs from TG minipigs (*n* = 5) (Fig. 2C). The Flp‐ERT2 recombinase mRNA was only observed in intestinal cells, confirming the intestinal specificity of the used villin promoter, while RFP expression from the oncogene cassette was observed in all organs.

### 3.3 Flp recombinase activates the oncogene cassette *in vitro*

**Figure 3:**
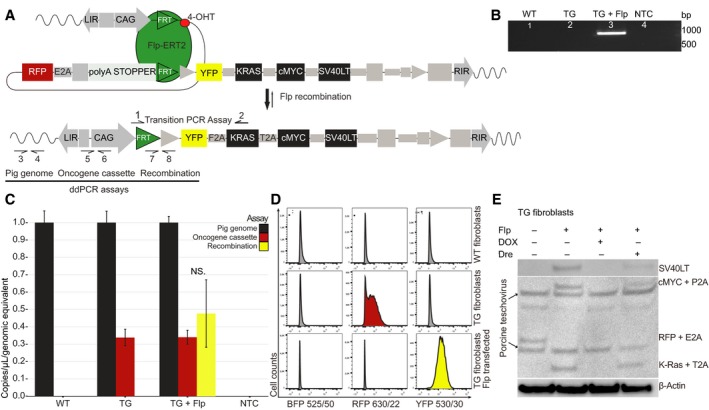
*In vitro* activation of the oncogene cassette through CAG‐Flp transfection. (A) Schematics of Flp‐mediated activation of oncogene cassette. Arrows indicate primers for the transition PCR assay (1–2) and for the three ddPCR assays; pig genome (3–4), oncogene cassette (5–6), and recombination (7–8); (B) validation of recombination in Flp‐transfected TG fibroblasts using the transition PCR assay. The expected transition band of 972 bp is seen in Flp‐transfected TG fibroblasts, but not in untransfected, or WT fibroblasts; (C) ddPCR quantification of pig genomes, oncogene cassettes, and recombination events in WT, TG‐ and TG CAG‐Flp‐transfected fibroblasts. Plotted are mean ± SE, normalized to pig genome equivalents for three biological replicates; (D) flow cytometric assessment of BFP, RFP, and YFP fluorescence from WT, TG‐ and TG CAG‐Flp‐transfected pig fibroblasts (*n* > 10 000 cells in each analysis). All fibroblasts are BFP negative as expected. There is a complete shift from RFP to YFP in TG fibroblasts upon Flp transfection. Shown is a representative example of three independent experiments; (E) western blotting of untreated TG fibroblast, CAG‐Flp‐transfected TG fibroblasts with either DOX treatment or subsequent CAG‐Dre recombinase transfection. Anti‐2A linker peptide antibody was used to visualize multiple protein expression with β‐actin as loading control. The anti‐2A linker peptide antibody also recognizes various porcine teschovirus proteins, which results in a few unspecific bands in the immunoblot.

Primary TG minipig (*n* = 3) fibroblasts were isolated from fetal ear notch biopsies, cultivated, and transfected with a CAG‐Flp activator construct to simulate intestinal Flp activation of the oncogene cassette (schematized in Fig. 3A). Conventional and droplet digital PCR (ddPCR) confirmed Flp recombinase‐mediated excision of the blocking element from the oncogene cassette (Fig. 3B, C). ddPCR further indicated that the majority of oncogene cassettes (95.2%) in the sample had been activated (Fig. 3C). This finding was corroborated by flow cytometry, which showed a complete shift from RFP to YFP and no nonspecific tissue BFP expression (Fig. 3D). A complete shift from RFP to YFP was also observed by fluorescence microscopy (Fig. S5). As expected, no BFP was observed in the villin‐negative fibroblasts. The activated oncogene unit produced similar levels of SV40LT, cMYC, and KRAS protein, and showed no sign of RFP expression (Fig. 3E). DOX treatment of the Flp recombined TG fibroblasts activated the rtTR‐KRAB and inhibited all expression from the oncogene unit (Fig. 3E) including removal of YFP emission (Fig. S5). Transfection with a Dre recombinase, which can mediate excision of the entire oncogene cassette, led to reduced oncogene protein levels (Fig. 3E). The fact that protein remained could indicate that the oncogene cassette was not excised in all cells.

### 3.4 4‐OHT treatment of TG intestinal cells activates the oncogene cassette *in vitro* and *ex vivo*

**Figure 4:**
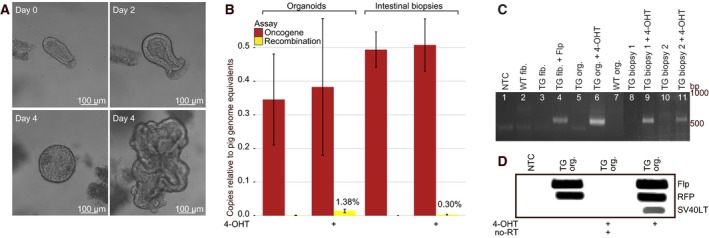
4‐OHT activation of the oncogene cassette in TG intestinal organoids (*in vitro*) and intestinal TG biopsies (*ex vivo*). (A) TG porcine intestinal organoids at days 0, 2, and 4 (with two distinct growth patterns); (B) quantification of oncogene cassettes and recombination events by ddPCR in 4‐OHT‐treated and untreated organoid cultures and intestinal biopsies. Shown are results of five to six biological replicates plotted as mean ± SE normalized to pig genome equivalents; (C) recombination validated by transition PCR in WT and TG organoid cultures (lanes 5–7) and intestinal biopsies (lanes 8–11). The transition PCR assay revealed recombination in all TG organoids and biopsies exposed to 4‐OHT, but in none of the untreated biopsies. NTC, WT fibroblasts, and TG fibroblasts +/− CAG‐Flp were included as controls (lanes 1–4); (D) Flp, RFP, and SV40LT transcript levels in TG organoids +/− 4‐OHT quantified by RT‐PCR.

To assess whether 4‐OHT treatment activates the oncogene cassette, organoid cultures were established from intestinal biopsies from TG minipigs (*n* = 5) and treated with 1 μm 4‐OHT upon crypt formation. Flp recombination of the oncogene cassette was confirmed in all 4‐OHT‐treated cultures (Fig. 4A–D). Using ddPCR, it was estimated that ~ 1.38% (1/27) of oncogene cassettes were recombined. Nonetheless, the induced SV40LT mRNA expression could easily be detected, indicating that the CAG promoter ensures high transcriptional activity (Fig. 4D). The RFP level was largely unchanged, as expected with only 1 of 27 oncogene cassettes recombined. Activation of the oncogene cassette was also tested *ex vivo*. Fresh colonic and rectal biopsies from TG minipigs (*n* = 6) were treated with 1 μm 4‐OHT. Recombination was seen in all treated biopsies at rates ranging from 0.04 to 0.74% (Fig. 4B,C). This confirmed that the oncogene cassette could be activated in both colonic epithelium and rectal epithelium.

### 3.5 Metastatic cancer *in vivo*

**Figure 5:**
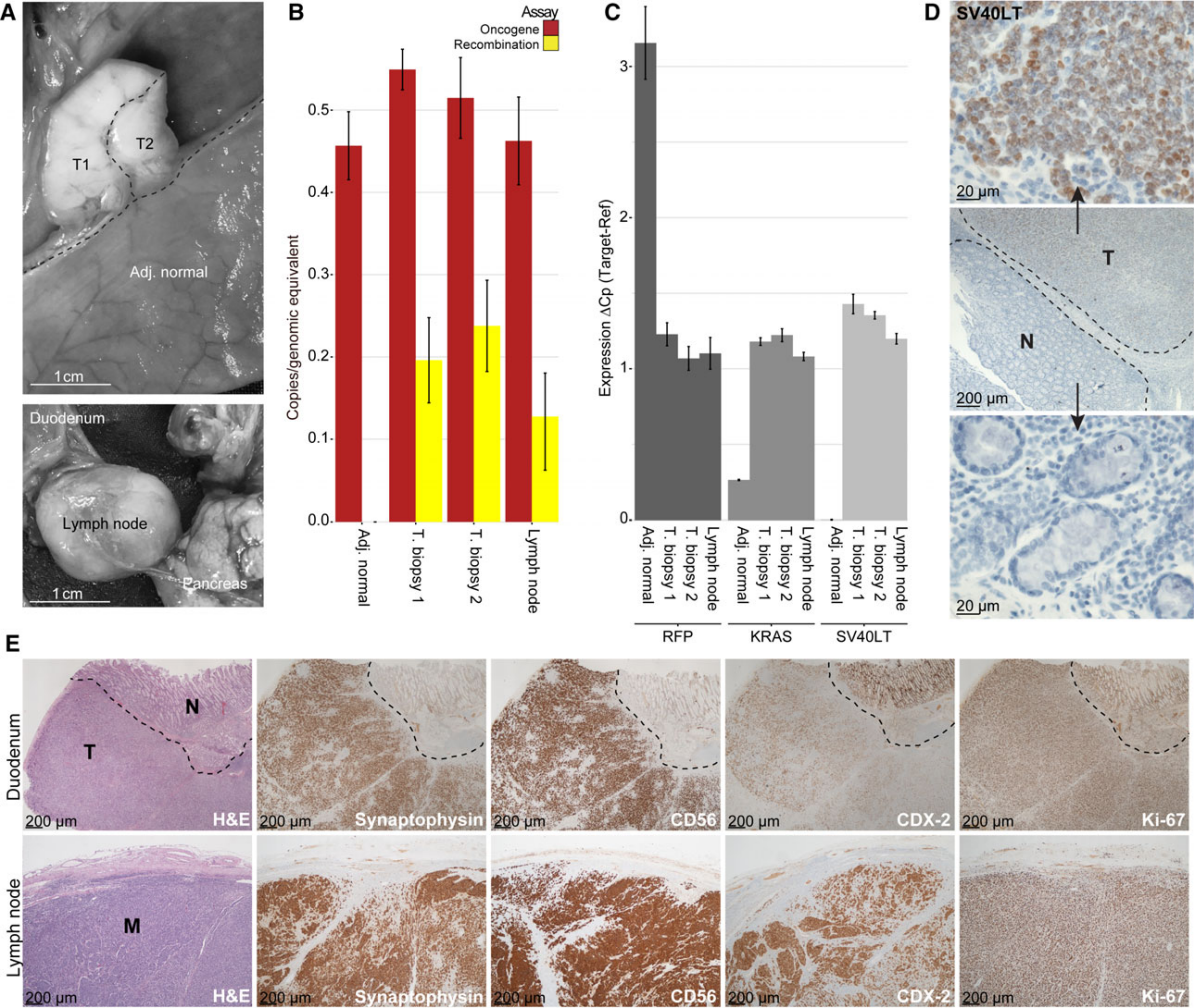
*In vivo* carcinogenesis after systemic tamoxifen treatment. (A) A duodenal tumor and an enlarged local lymph node were found in the resected specimen; (B) quantification of the number of oncogene cassettes and recombination events by ddPCR in adjacent normal duodenal epithelium and biopsies of the tumor (T1 and T2) and lymph node; (C) RFP, KRAS, and SV40LT transcription levels in adjacent normal tissue, carcinoma, and lymph node, quantified by RT‐PCR; (D) immunohistochemistry reveals the carcinoma cells to be SV40LT positive; (E) H&E and IHC for the duodenal tumor and lymph node metastasis with the neuroendocrine differentiation markers synaptophysin and CD56, as well as the intestinal epithelial marker CDX‐2 and the proliferation marker Ki‐67.

Encouraged by our ability to activate the oncogene cassette in intestinal biopsies *ex vivo*, systemic tamoxifen treatment was initiated. Tamoxifen was administered perorally to TG minipigs (*n* = 3) (400 mg per day) for 2 × 5 days with a 7‐day break in between. Two pigs were sacrificed 72 days after treatment initiation; a duodenal tumor with metastatic spread to a local lymph node was detected in one of the pigs (Fig. 5A). The other pig showed no signs of tumor growth or activation of the oncogene cassette, neither did the third pig that was sacrificed 218 days after treatment initiation (data not shown). ddPCR analysis of the duodenal tumor and lymph node metastasis showed that 30–50% of the oncogene cassettes were activated (Fig. 5B). Given that all TG cells have one oncogene cassette, this indicates that 50–70% of the cells in the tumor lesions are of nonepithelial origin or normal unactivated epithelial cells. Consistent with activation, and excision of the blocker element (including the RFP reporter) in cancer cells, the RFP transcript level in biopsies from tumor and metastasis was reduced 0.38‐fold (95% CI: 0.36–0.40) compared to adjacent normal tissue (Fig. 5C). By contrast, the transcript levels of the oncogene KRAS was increased 4.36‐fold (95% CI: 4.20–4.53), and *de novo* SV40LT transcription was detected. Exogenous SV40LT protein was consistently found in tumor cells (100%), but not in adjacent normal epithelia and connective tissue (Fig. 5D). The cancer cells in the duodenal tumor and lymph node both expressed the epithelial marker CDX2, as well as the neuroendocrine markers CD56, and synaptophysin (Fig. 5E), indicating that the metastasis originated from the duodenal tumor. The neuroendocrine markers were highly expressed in the membrane and cytoplasm in the majority of the primary tumor (85–90%), while the epithelial marker CDX2 was focally positive in 60% of the primary tumor. The same staining patterns were seen in the lymph node metastasis (Fig. 5E). Histopathology was assessed blinded by an experienced pathologist and showed invasion into muscularis propria, indicating a pT2, pN1 large cell neuroendocrine tumor (NET). The proliferation marker KI67 was positive in roughly 90% of the carcinoma and metastasis (Fig. 5E). There were no signs of tumor formation in the remaining treated pigs (*n* = 2), in WT pigs treated with the same tamoxifen dose (*n* = 9), or in untreated TG pigs (*n* = 2).

## 4 Discussion

In this study, we have successfully generated an inducible oncopig model. The study was a small (*n* = 3) proof‐of‐principle study with the primary goal of inducing intestinal cancer. Upon systemic treatment with tamoxifen, we observed a carcinoma with lymph node metastasis already 2 months post‐treatment in one of three pigs. Animal models are important tools for cancer research. Pigs resemble humans in regard to, for example, size, physiology, and lifespan. Although pigs are more costly and require more space than rodent models, results from studies of pigs might be easily translated directly to the clinic. Unlike pig models for spontaneous cancer that require long time for cancer development (Duran‐Struuck *et al*., 2010), the model presented here developed metastatic disease only two months after activation. The large size of pigs and their overall similarity to humans open up for novel *in vivo* experiments of intestinal cancer, enabling treatment response studies, with longitudinal sample collection from the primary tumor. The present model is inducible and, compared to spontaneous models, highly controllable. In principle, it should be possible to target the intestinal epithelium directly by delivery of 4‐OHT through endoscopy, potentially opening up for parallel initiation of multiple tumors allowing for synchronous replicates in different areas of the intestine in the same pig.

As an initial *in vivo* test of the model, we used systemic tamoxifen treatment. While one might have expected that this would cause tumors throughout the intestine, we observed a single tumor only, located in the small intestine of one of the pigs. We speculate that this may be due to either a low activation rate, for example, due to a low 4‐OHT concentration in the mucosal layer, or that activation of the oncogene cassette does not induce sufficient stem cell properties, meaning that activated differentiated cells continue to migrate up the crypt–villus axis for eventually to be exfoliated. This would mean that only if the activated cell is a stem cell, it is likely that a tumor will be formed. The low frequency of recombination observed in the organoids and treated biopsies indicates that only few cells are activated either due to low 4‐OHT accessibility, due to low efficiency of the 4‐OHT‐activated Flp‐ERT2 recombinase, or due to a high fraction of the cells in the biopsies being nonintestinal cells (which do not express Flp‐ERT2) incapable of activation. Another explanation could be that oncogene cassette activation in intestinal cells leads to apoptosis. It has been reported that high expression of KRAS and cMYC, as well as inhibition of p53/pRb, can induce apoptosis (Podsypanina *et al*., 2008). We did not observe any activation in the adjacent normal mucosa, supporting the view that either activation levels are low or activated cells have died or been exfoliated during the two‐month period from 4‐OHT treatment until the pigs were sacrificed. One or more of these mechanisms potentially also explain why only one of three 4‐OHT‐treated TG minipigs developed cancer. We further speculate that the localization of the tumor in the small intestine is a consequence of the systemic treatment, where a higher concentration of 4‐OHT is to be expected, due to tamoxifen being metabolized in the liver and partly secreted as the active metabolite 4‐OHT through the bile (Fromson *et al*., 1973). Characterization of the duodenal tumor showed that it was of epithelial origin and followed the neuroendocrine differentiation lineage. Unlike human duodenal NETs, this tumor was highly malignant and produced lymph node metastasis within 2 months. Perhaps this was expected as the oncogenesis in this model is driven by overexpression of cMYC, KRAS‐G12D, and SV40LT, of which the latter inhibits the tumor suppressors p53 and pRB. This combination is rarely observed in human duodenal NETs, but in contrast is frequently observed in the more aggressive NETs and other carcinomas of the colon (Kleist *et al*., 2014), raising the concern that the presented model might not fully reflect the etiology of human intestinal cancers. However, our rationale for deregulating four of the most potent cancer pathways (cMYC, KRAS, p53, and pRB) was primarily to increase the likelihood of fast development of full‐blown tumors. Additional studies using more oncopigs are needed to characterize the prevalence and biology of the tumors produced, and their relation to human cancer.

Until now, no pig model has been reported to produce intestinal carcinomas. A recent study reported an APC‐mutated pig (Flisikowska *et al*., 2012), which yielded multiple adenomas in the colon; however, none developed into carcinomas. Introducing a latent oncogenic mutated p53 into transformed porcine mesenchymal cells leads to *in vivo* lymphoma, renal, and osteogenic tumors (Leuchs *et al*., 2012; Sieren *et al*., 2014). Likewise, a porcine Cre‐inducible model with p53 and KRAS mutations (Schook *et al*., 2015) described that tumors of mesenchymal origin developed at AdCre injection sites. The advantage of our model is the direct targeting of intestinal epithelia through the villin‐controlled Flp‐ERT2 expression. This is clinically more relevant than a model where any cell type has the potential of transforming into cancer upon injection of the activating agent. Note, however, that the AdCre model by Schook *et al*., as well as our pig model in principle, can be activated tissue specifically if the delivered recombinase (Flp or Cre) is controlled by a tissue‐specific promoter. Using such an approach, the oncogene cassette could potentially be activated in any organ, including pancreas and lung where KRAS and p53 often are deregulated in tumors.

We foresee great potential for the presented model, particularly if an approach for targeted initiation of carcinogenesis is established, for example, by injections of 4‐OHT directly into the intestinal wall. It will be critical to ensure sufficiently high concentrations of 4‐OHT and long enough retention time of the drug for the activation of sufficient numbers of intestinal stem cells at the injection site. ERT2‐inducible murine models are commonly activated by intraperitoneal injection of 4‐OHT oil solutions. However, in minipigs, such approach would require large quantities of 4‐OHT and the risk of hazardous oil‐induced inflammation (Marques da Silva *et al*., 2008; Ziv *et al*., 2015). Alternative delivery systems could involve electroporation, osmotic pumps, and/or 4‐OHT‐loaded nanoparticles (Devulapally *et al*., 2015). Tumor initiation in the distal colon would allow easy access to collect biopsies through endoscopies enabling longitudinal studies. Such studies would allow cancer progression and clonal evolution to be studied over time both in the absence and in the presence of therapy‐induced selection. Such longitudinal studies are not possible in humans, where it is unethical not to remove the primary tumor.

In summary, we have generated the first inducible oncopig model of intestinal cancer. As discussed above, the current interpretation of the study is limited by several factors, most notably (a) a low number of animals used; (b) a single event of successful carcinogenesis; (c) the unknown relation between the observed NET tumor and the biology of human intestinal cancers; (d) the lack of control of 4‐OHT delivery when given systemically. However, the model also provides a range of novel and unique possibilities to explore intestinal carcinogenesis from initiation and all the way to metastasis.

## Author contributions

JEJ, MMC, MHR, TFØ, and CLA were responsible for study concept and design. YL, RL, and HC performed the cloning. MMC, SSA, IL, MWØ, JEJ, SH, FDH, KBJ, PJS, SL, MFB, and MKT performed the experiments and involved in the acquisition of the data. MMC, SSA, IL, MWØ, JEJ, SH, MKT, MHR, and CLA took part in the analysis and interpretation of the data. MMC, SSA, and CLA drafted the manuscript. All authors critically reviewed and approved the final version of the manuscript.

## Supporting information


**Fig. S1.** Tissue specificity of the villin promoter.

**Fig. S2.** Selection of TG clones for SCNT.

**Fig. S3.** Overview of the 13 farrowed oncopigs and the results of the study.

**Fig. S4**. IVIS scans of full organ panels from WT and TG pigs.

**Fig. S5.** Primary TG fetal fibroblasts transfected with Flp recombinase and subsequently DOX treated for five days (750 ng·mL^−1^).

**Table S1**. Primers used for QPCR, LDI‐PCR, RT‐qPCR, transition PCR, genomic PCR, and ddPCR.
