# Supplementary figures and images for: A genetically inducible porcine model of intestinal cancer
Source: Mol Oncol. 2017 Oct 10;11(11):1616–29. doi: 10.1002/1878-0261.12136 (PMC5664002; doi:10.1002/1878-0261.12136)

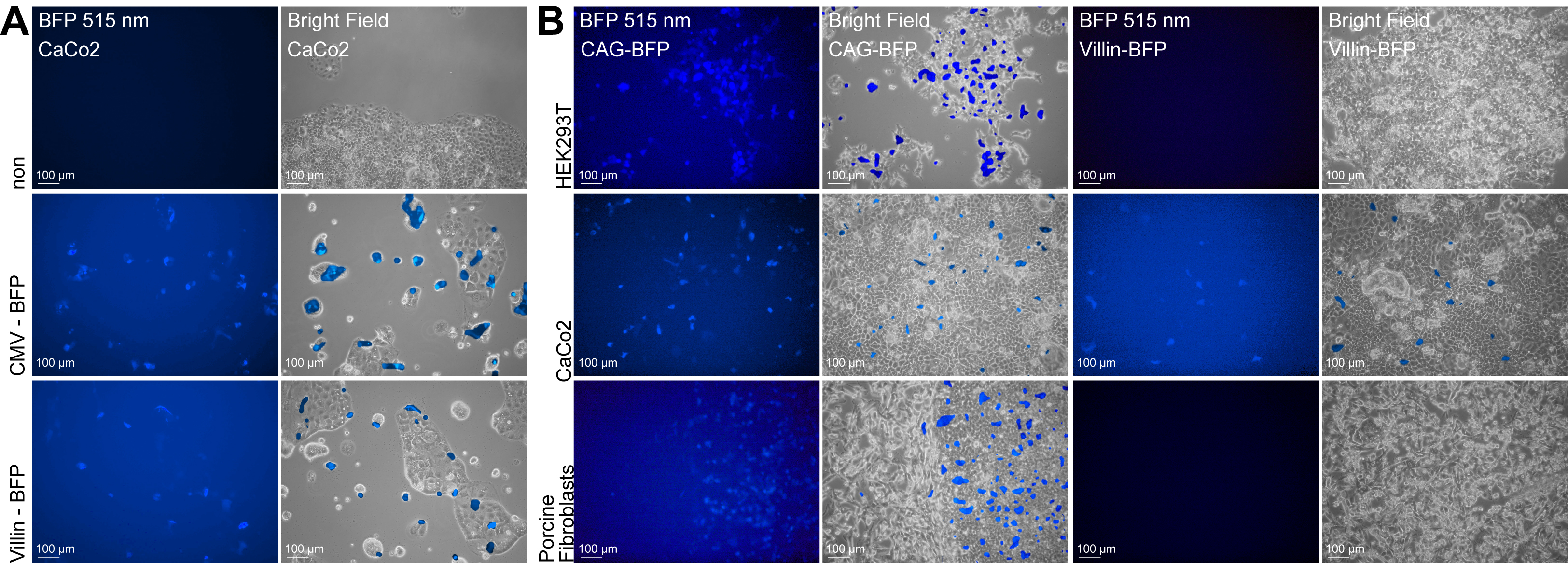

Supplement: Supplementary file 1 — Fig. S1. Tissue specificity of the villin promoter. [file MOL2-11-1616-s001.jpg]

**A**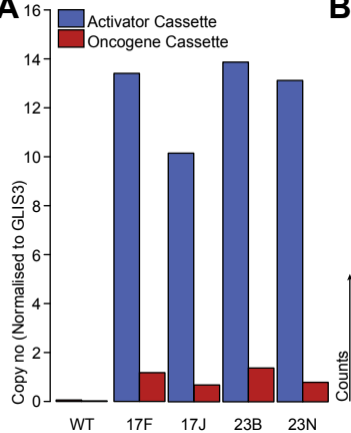**B**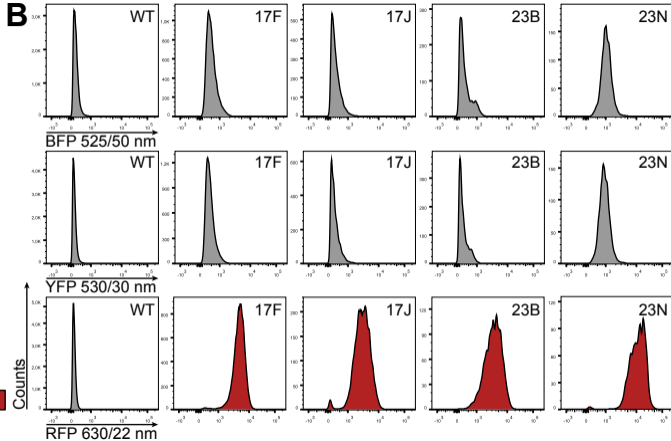**C**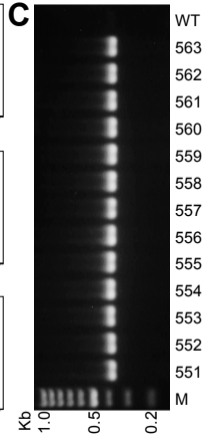

Supplement: Supplementary file 2 — Fig. S2. Selection of TG clones for SCNT. [file MOL2-11-1616-s002.pdf]

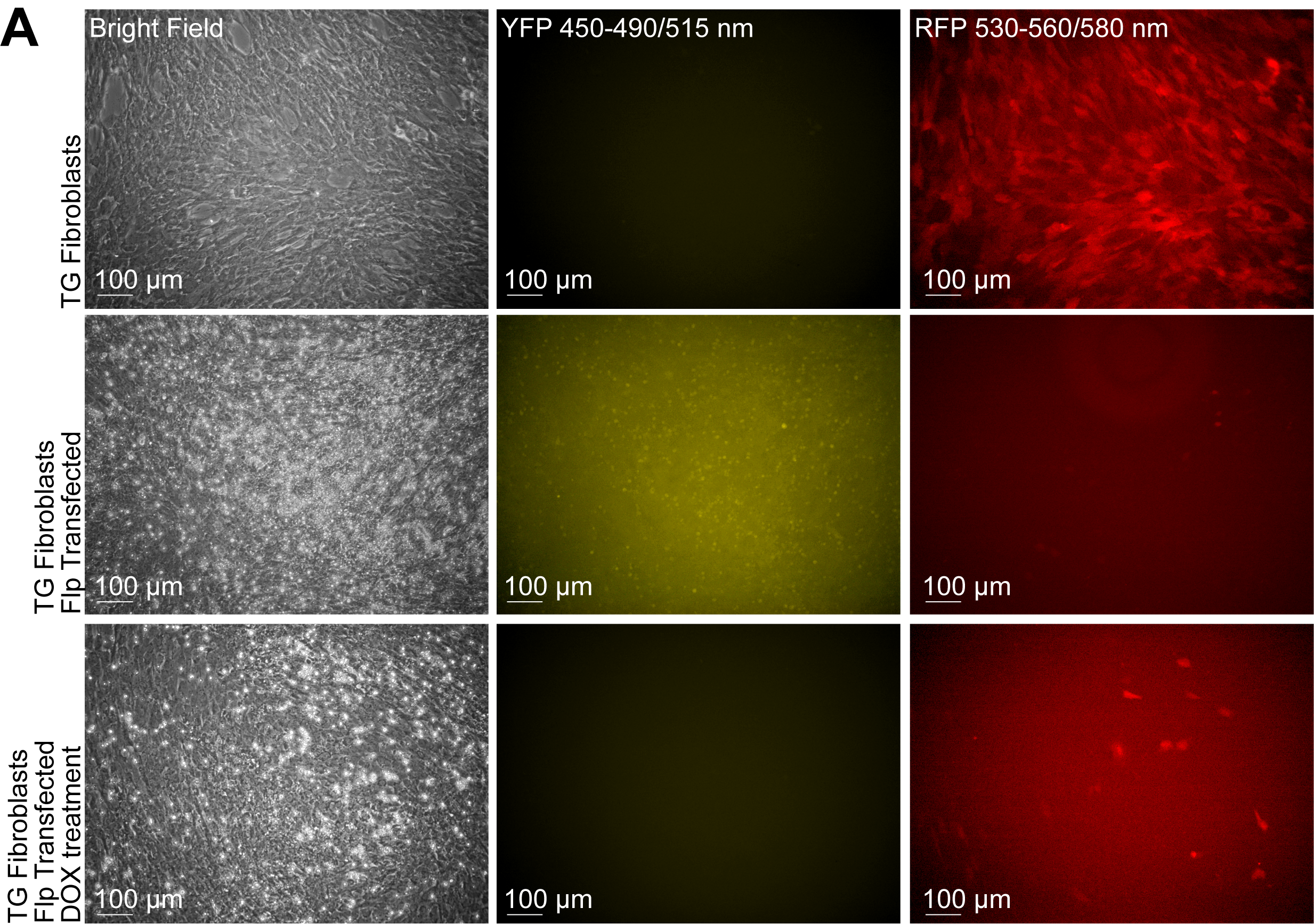

Supplement: Supplementary file 5 — Fig. S5. Primary TG fetal fibroblasts transfected with Flp recombinase and subsequently DOX treated for five days (750 ng·mL−1). [file MOL2-11-1616-s005.png]
