# Supplementary material for: A genetically inducible porcine model of intestinal cancer
Source: Mol Oncol. 2017 Oct 10;11(11):1616–29. doi: 10.1002/1878-0261.12136 (PMC5664002; doi:10.1002/1878-0261.12136)
Supplement: Supplementary file 3 — Fig. S3. Overview of the 13 farrowed oncopigs and the results of the study. [file MOL2-11-1616-s003.pdf]

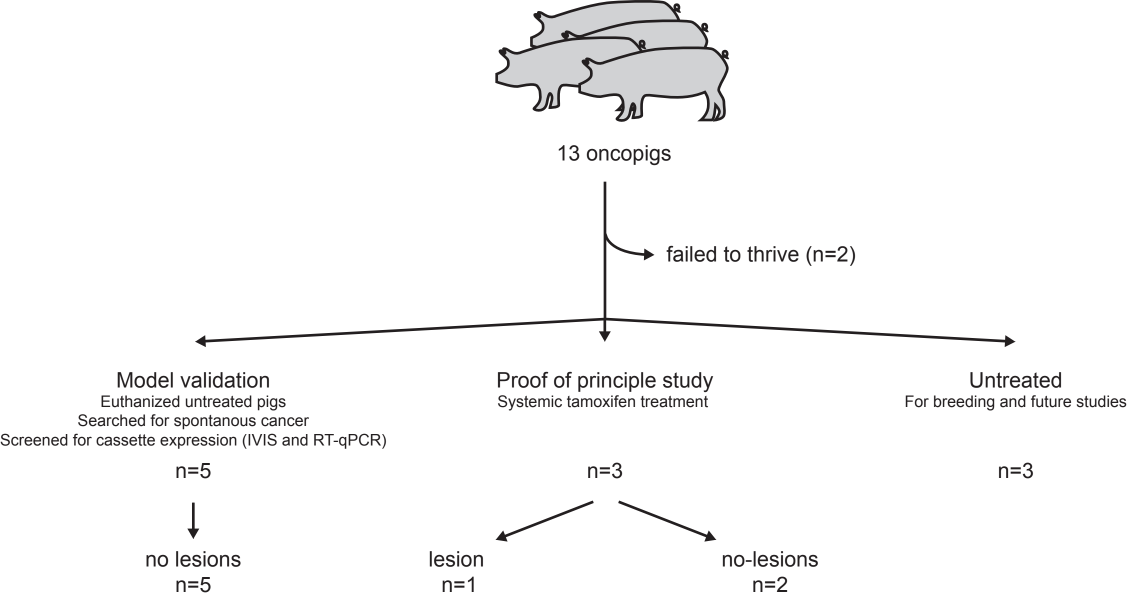
13 oncopigs
failed to thrive (n=2)
Model validation
Euthanized untreated pigs
Searched for spontanous cancer
Screened for cassette expression (IVIS and RT-qPCR)
n=5
Proof of principle study
Systemic tamoxifen treatment
n=3
Untreated
For breeding and future studies
n=3
no lesions
n=5
lesion
n=1
no-lesions
n=2
