# Supplementary material for: A genetically inducible porcine model of intestinal cancer
Source: Mol Oncol. 2017 Oct 10;11(11):1616–29. doi: 10.1002/1878-0261.12136 (PMC5664002; doi:10.1002/1878-0261.12136)
Supplement: Supplementary file 6 — Table S1. Primers used for QPCR, LDI‐PCR, RT‐qPCR, transition PCR, genomic PCR, and ddPCR. [file MOL2-11-1616-s006.docx]

**Supplementary Table 1** Primers used for Q-PCR, LDI-PCR, RT-qPCR, transition PCR, genomic-PCR, and ddPCR.

| **PCR method** | **Assay** | **Forward primer (5’-3’)** | **Reverse primer (5’-3’)** | **Probe (5’-3’)** |
| --- | --- | --- | --- | --- |
| **Copy no. qPCR** | GLIS3 | CCCACACTACCCTGACTCT | TGTAATGCCCGAGTGAGTTG |  |
|  | RFP | CCTGGCTACCAGCTTCATGT | GTGGTGATCCTCTCCCATGT |  |
|  | BFP | CACCATCTTCTTCAAGGACG | AGTTGTACTCCAGCTTGTGC |  |
| **LDI-PCR** | Primary | CAGCCATTGCCTTTTATGGT | AAATACAAAATTGGGGGTGG |  |
|  | Nested | GCTGGTTGTTGTGCTGTCTC | GGGCGTACTTGGCATATGAT |  |
| **RT-qPCR** | RFP | CCTGGCTACCAGCTTCATGT | GTGGTGATCCTCTCCCATGT |  |
|  | Flp | CCGAGAAGATCCTGAACAGC | TCTTGATGTCGCTGAACCTG |  |
|  | RPL4 | CAAGAGTAACTACAACCTTC | GAACTCTACGATGAATCTTC |  |
|  | HPRT1 | GGACTTGAATCATGTTTGTG | CAGATGTTTCCAAACTCAAC |  |
|  | TBP | AACAGTTCAGTAGTTATGAGCCAGA | AGATGTTCTCAAACGCTTCG |  |
| **Transition PCR** | Cassette (976bp)  (No. 1/2) | GCTGGTTGTTGTGCTGTCTC | ACGCCATCAGCTCCAACTAC |  |
| **Genomic PCR** | Oncogene cassette integration (420bp) | CTCTTCGCCCTCCCAGTTTA | TCGCGACTGTTTAAAGGCAC |  |
| **ddPCR** | Pig Genome (No. 3/4) | CCTCGAGGTATAAAACAGCATT | CTCTGTCATGGGAGACTCCTT | TTGACTTTCTTTAGCGGAATGTCAA |
|  | Cassette (No. 5/6) | ATATGGAGTTCCGCGTTACA | TGGCGTTACTATGGGAACAT | TTACGGTAAATGGCCCGCCT |
|  | Recombination FRT (No. 7/8) | TGGTTGTTGTGCTGTCTCAT | CGCGATCGCAATAACTTT | AGGAACTTCCTCATGCGCAT |
