# Supplementary material for: A genetically inducible porcine model of intestinal cancer
Source: Mol Oncol. 2017 Oct 10;11(11):1616–29. doi: 10.1002/1878-0261.12136 (PMC5664002; doi:10.1002/1878-0261.12136)
Supplement: Supplementary file 4 — Fig. S4. IVIS scans of full organ panels from WT and TG pigs. [file MOL2-11-1616-s004.pdf]

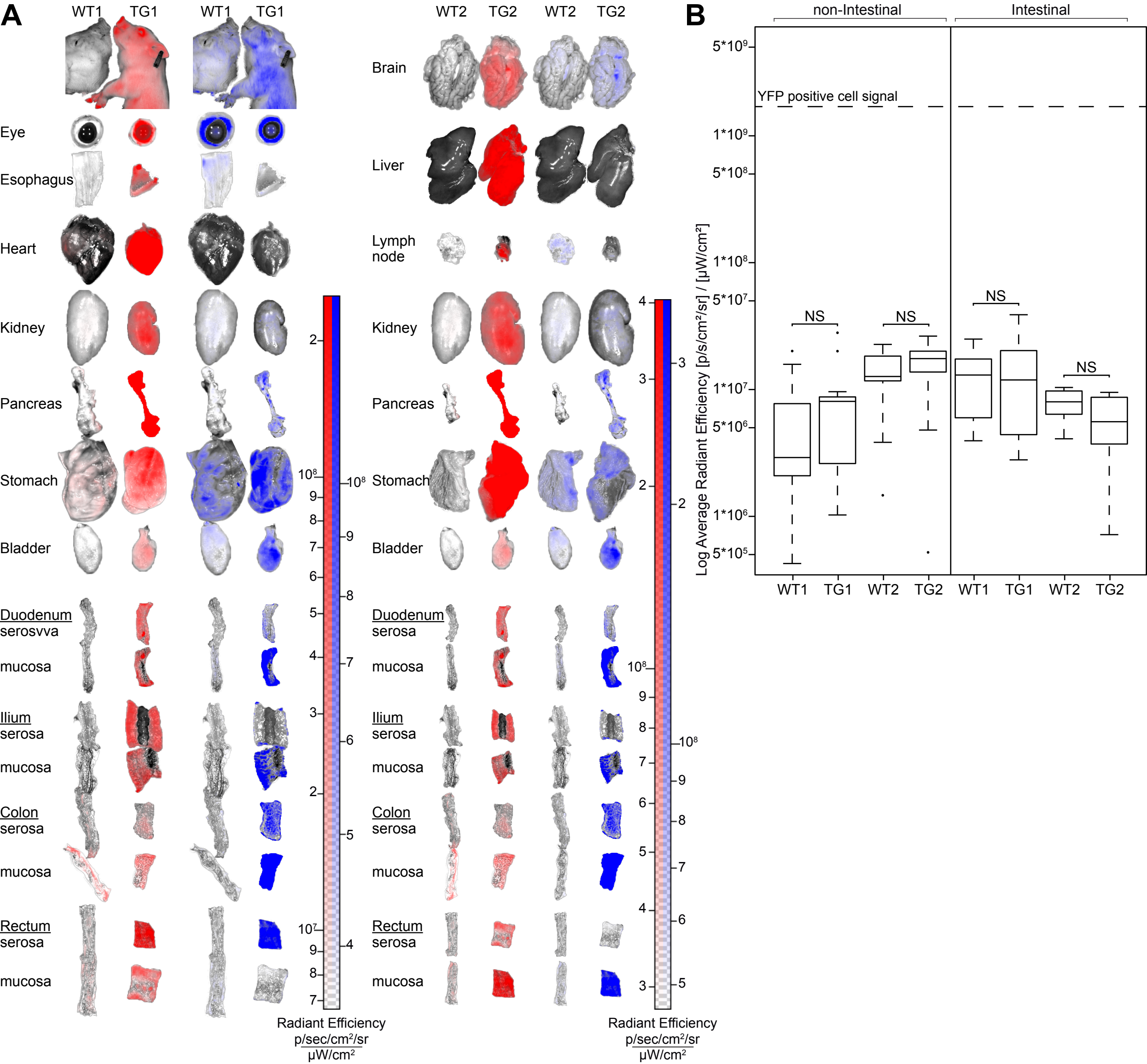
A
WT1
TG1
WT1
TG1
WT2
TG2
WT2
TG2
Eye
Esophagus
Heart
Kidney
Pancreas
Stomach
Bladder
Duodenum
serosvva
mucosa
Ilium
serosa
mucosa
Colon
serosa
mucosa
Rectum
serosa
mucosa
Brain
Liver
Lymph node
Kidney
Pancreas
Stomach
Bladder
Duodenum
serosa
mucosa
Ilium
serosa
mucosa
Colon
serosa
mucosa
Rectum
serosa
mucosa
Radiant Efficiency
p/sec/cm²/sr
μW/cm²
Radiant Efficiency
p/sec/cm²/sr
μW/cm²
B
non-Intestinal
Intestinal
YFP positive cell signal
Log Average Radiant Efficiency [p/s/cm²/sr] / [μW/cm²]
5*10⁹
1*10⁹
5*10⁸
1*10⁸
5*10⁷
1*10⁷
5*10⁶
1*10⁶
5*10⁵
NS
NS
NS
NS
WT1
TG1
WT2
TG2
WT1
TG1
WT2
TG2
